# Numerical Investigation of Ultrashort Laser-Ablative Synthesis of Metal Nanoparticles in Liquids Using the Atomistic-Continuum Model

**DOI:** 10.3390/molecules25010067

**Published:** 2019-12-24

**Authors:** Dmitry S. Ivanov, Thomas Izgin, Alexey N. Maiorov, Vadim P. Veiko, Baerbel Rethfeld, Yaroslava I. Dombrovska, Martin E. Garcia, Irina N. Zavestovskaya, Sergey M. Klimentov, Andrei V. Kabashin

**Affiliations:** 1 Department of Physics and OPTIMAS Research Center, TU Kaiserslautern, 67663 Kaiserslautern, Germany; rethfeld@physik.uni-kl.de; 2 Institute of Physics and Center for Interdisciplinary Nanostructure Science and Technology (CINSaT), University of Kassel, 34125 Kassel, Germany; thomas.izgin@hotmail.de (T.I.); m.garcia@uni-kassel.de (M.E.G.); 3 Institute of Engineering Physics for Biomedicine (PhysBio), MEPHI, 115409 Moscow, Russia; anmaiorov@vega.su (A.N.M.); yara.dombrovska@gmail.com (Y.I.D.); INZavestovskaya@mephi.ru (I.N.Z.); kliment-61@mail.ru (S.M.K.); 4 Physics Department, ITMO University, 197101 St. Petersburg, Russia; vadim.veiko@mail.ru; 5 P. N. Lebedev Physical Institute of Russian Acad. Sci., Leninskiy Pr. 53, 119991 Moscow, Russia; 6 LP3, Aix Marseille Univ, CNRS, LP3, Campus de Luminy, Case 917, 13288 Marseille, France

**Keywords:** pulsed laser ablation in liquids, femtosecond laser ablation, dual nanoparticle distribution, metal nanoparticles

## Abstract

We present a framework based on the atomistic continuum model, combining the Molecular Dynamics (MD) and Two Temperature Model (TTM) approaches, to characterize the growth of metal nanoparticles (NPs) under ultrashort laser ablation from a solid target in water ambient. The model is capable of addressing the kinetics of fast non-equilibrium laser-induced phase transition processes at atomic resolution, while in continuum it accounts for the effect of free carriers, playing a determinant role during short laser pulse interaction processes with metals. The results of our simulations clarify possible mechanisms, which can be responsible for the observed experimental data, including the presence of two populations of NPs, having a small (5–15 nm) and larger (tens of nm) mean size. The formed NPs are of importance for a variety of applications in energy, catalysis and healthcare.

## 1. Introduction

Pulsed laser ablation has emerged as a powerful technique for the synthesis of nanoparticles, which profits from a natural production of nanoclusters during laser–materials interaction [1,2]. When ablated in gaseous ambient, the nanoclusters can be deposited on a substrate to form a thin nanostructured film [3,4,5,6,7,8,9], while ablation in a water environment leads to the formation of a colloidal nanoparticle solution [10,11,12,13,14,15,16,17,18]. Such a synthesis can lead to exceptional purity of formed nanomaterials, while their properties are often unique and not reproducible by conventional chemical methods.

Pulsed laser ablation in liquids (PLAL) has demonstrated spectacular progress for last 15 years [19] due to its versatility and relative cost efficiency in the synthesis of a variety of nanomaterials. When laser-ablated nanoclusters are injected into water or a neutral organic environment, they cool down and gradually coalesce to form larger NPs, while the presence of chemically active species in the solution can stop their growth and decrease therefore the NPs size [12,20]. In the absence of ligands, the colloidal NPs are stable [16] and have bare (ligand-free) surface, which opens up prospects for a variety of applications in energy [21], catalysis [22] and healthcare [23,24]. As an example, we recently showed that bare Au-based NPs can provide one order of magnitude better electrocatalytic activity toward glucose oxidation compared to all chemically synthesized counterparts [25], prominent response SERS-based bioidentification tasks [26,27], as well as a much-reduced toxicity in biomedical applications [28,29].

**Figure 1:**
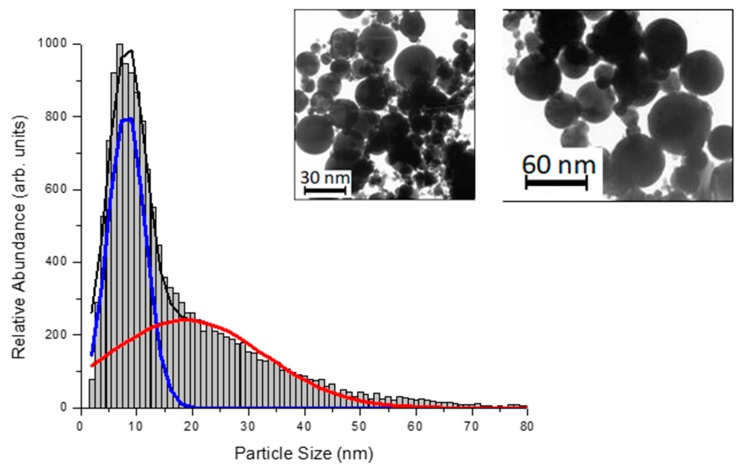
Typical size distribution of gold nanoparticles prepared by 300 fs PLAL (1025 nm) from a gold target in deionized water under the pulse energy of 2 J/cm^2^.

It is accepted that ultrashort (fs, ps) laser ablation appears to be especially efficient in the control of nanoparticle size in the absence of ligands [14,15]. This case is characterized by a much lower pulse energy to initiate ablation, which minimizes plasma and cavitation bubble effects [30] and thus makes possible a fine control NPs size parameters [14,18]. However, despite the collection of numerous data on PLAL synthesis of nanomaterials, a simple interpretation of all range of phenomena is still difficult. As an example, there is no common vision of mechanisms responsible for particular size properties of formed NPs. Here, bimodal size distributions of metal and other NPs (see an example in Figure 1) were typically reported under the use of radiation of different parameters [14,15,30,31,32,33,34,35], but under certain experimental conditions only one NP population was recorded [14,30,36]. The difficulty for a simple interpretation of data is obviously due to a variety of parameters and processes involved in PLAL synthesis. The formation of NPs is accompanied by a series of events, including radiation absorption, plasma generation and cavitation bubbles collapse [37]. These events can lead to both thermal [14,15,33] and mechanical [14,15] mechanisms of ablation, as well as instabilities at the plume–liquid interface [38]. In this sense, a development of a model, which could adequately describe thermal and mechanical effects and their interplay, could help to advance toward a better understanding and interpretation of the phenomena.

Here, we propose a model, combining the Molecular Dynamics (MD) and Two Temperature Model (TTM) approaches, in order to enable the modeling of ultrashort laser pulse interaction with metal targets [39]. This MD-TTM model was already successfully used in investigation of the mechanism of periodic nanostructuring of thin and thick metal targets by laser pulses of different wavelength [40,41]. In this paper, we further elaborate this model and extend it to simulations of NPs generation in liquid environment in order to clarify conditions and mechanisms of synthesis of metal (Au) NPs during the interaction of fs radiation with a solid target. As one of main results of this study, our calculations reproduced conditions for the bimodal NPs size distribution and this result was explained by a competitive contribution of thermal and cavitation explosion mechanisms that are referred to as thermal and mechanical damage of the target in the following theoretical portion of this paper.

## 2. Results and Discussions

### 2.1. Setting Up the Computational Cell

**Figure 2:**
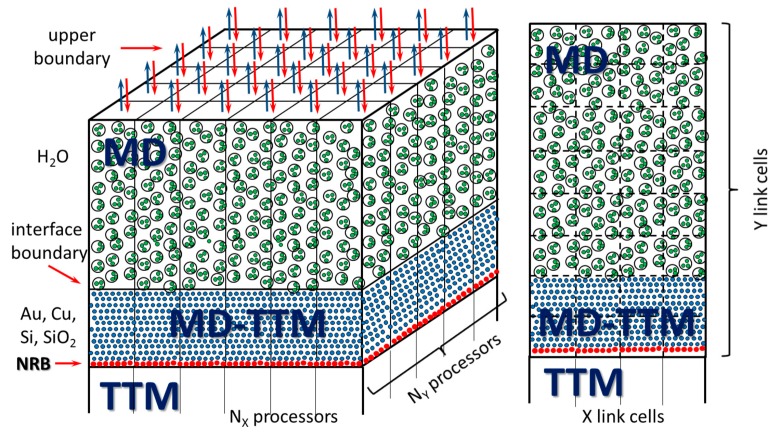
The computational cell is schematically shown for the case of modeling of the laser-induced processes under water confinement on the experimental scale using Message Passing Interface (MPI) multiprocessing.

The suggested atomistic-continuum (MD-TTM, see Section 3.2) approach has proven itself as a powerful numerical tool when studding the kinetics of ultrafast laser-induced non-equilibrium processes and can be used in the present research on investigation of the mechanisms of the laser-induced generation of NPs in water. The total computational cell is schematically shown in Figure 2.

The gold-water MD supercell consisting of ~185,000,000 atoms was taken with dimensions of 62 × 62 × 1250 nm^3^ in X, Y, and Z directions, respectively, with the thicknesses of 250 nm for the metal and 1000 nm for the water layers with the atomic resolution. In order to avoid unnecessary MD simulations in deep layers of gold material, at a certain depth (>250 nm) from the surface, where no phase transformation can occur, we imposed the Nonreflective Boundary (NRB) conditions. Demanded by the investigated physics, the MD-TTM model was applied only above that limit. The dynamically behaving NRB conditions are aimed to absorb the incoming laser-induced pressure wave and they are transparent for the heat flux. The ordinary TTM model was solved beneath the NRB taking into account the electron and phonon temperature dynamics on a scale up to 50 µm beneath the irradiated surface. By way of analogy, the NRB boundaries atop the water layer mimic the infinitely thick water layer and placed at a distance of 1000 nm, above which only mechanical action of water is accounted for. In each particular processor core, an atomistic-continuum MD-TTM model for metal part and ordinary MD model for water part are solved in 3D space (internal mesh is shown for one processor core, Figure 2).

For the direct comparison of the simulations result with the experiment, the MD-TTM model was implemented using a realistic interatomic potential for gold [42]. This potential reproduces the experimental thermophysical properties of the modeled material (such as equilibrium melting temperature, heat capacity, heat of fusion, volume of melting, and linear thermal expansion coefficient) with an accuracy of more than 99.5%. Furthermore, in this work we used a newly designed interatomic potential for water by Zhakhovsky, described in [43], and based on general concepts of the possible laser-induced mechanisms involved into the formation of NPs. This potential treats the water molecule as a single particle, but represents its mechanical properties with a high accuracy of more than 99%, which gives us the possibility for the quantitative comparison of the simulation results with the experimental data as soon as the effect due to chemical reactions (oxidation and dissociation) can be neglected. Based on the assumptions above, the suggested computational approach focuses on the identification of the general effects involved into the NPs formation taking into account the only mechanical action of water. Such a general approach, however, is an important step forward towards the understanding of the physics involved into the laser-generated NPs in liquid media. A similar approach, isolating the complex dynamics of the laser-generated processes, was already successfully implemented in the investigation of the essential mechanisms responsible for the nanostructuring process in [44].

### 2.2. Simulation Results and Discussions

**Figure 3:**
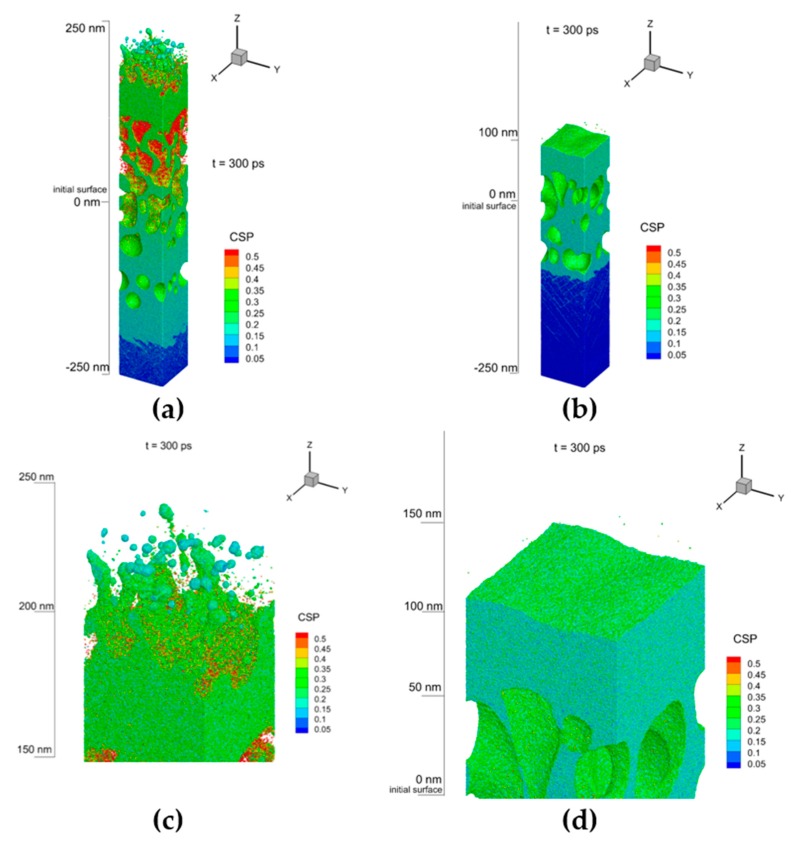
The atomic snapshots are shown for the process of short laser pulse nanoparticles generation in water for gold at the time of 300 ps from the beginning of simulations. The pulse duration of 0.3 ps was applied in (**a**) and the top region is zoomed for a better visual analysis in (**b**). The pulse duration of 5.0 ps was applied in (**c**) and the top region is zoomed for a better visual analysis in (**d**). The atoms are colored by Central Symmetry Parameter (CSP) for identification of their local atomic structure as follow: solid < 0.08 < defects < 0.12 < liquid < 0.25 < surface < 0.50 < vapor. The water atoms are blanked here for visualization of metallic part of the systems.

In order to extract the essential mechanisms responsible for the NPs formation in liquids during short laser pulse interactions with metal targets, we performed, analyzed, and compared the simulations for two different laser pulse durations—0.3 ps and 4.0 ps—at the same incident fluence of 2 J/cm^2^. The results of simulations of both cases are shown in Figure 3 (a,b) and (c,d) as atomic snapshots taken at the time of 300 ps for the shorter and the longer pulses correspondingly. For a better visual analysis, the water atoms are blanked and Figure 3 contains the zoomed top areas (b) and (c) of the corresponding cases. The atoms are colored by Central Symmetry Parameter (CSP) for identification of their local structure: solid < 0.08 < defects < 0.12 < liquid < 0.25 < surface < 0.50 < vapor.

Figure 3 exposes a great difference in the results of NPs generation with the laser pulses of different durations. If for the case of 0.3 ps pulse duration, we can observe the process of active formation of the NPs of 5–70 nm size, for the pulse of 4.0 ps, the NPs are absent for the same simulation time. Despite the fact that the same incident fluence of 2 J/cm^2^ was applied for both pulse durations of 0.3 ps (Figure 3a,b), and 4.0 ps (Figure 3c,d), the apparent difference between two cases can be attributed to the lower intensity in W/cm^2^ for the longer pulse and the lower values of the absorbed laser energy as a result. This difference originates from the dynamically changing reflectivity function versus electronic temperature at the metal surface [45]. The shorter pulse duration triggers higher values of the electronic temperature that results in a better absorption of laser energy (*R* = 0.843) with the absorbed fluence F_abs_ = 361 mJ/cm^2^, whereas the longer pulse leaves the reflectivity vale high (*R* = 0.930) with the absorbed fluence F_abs_ = 165 mJ/cm^2^.

**Figure 4:**
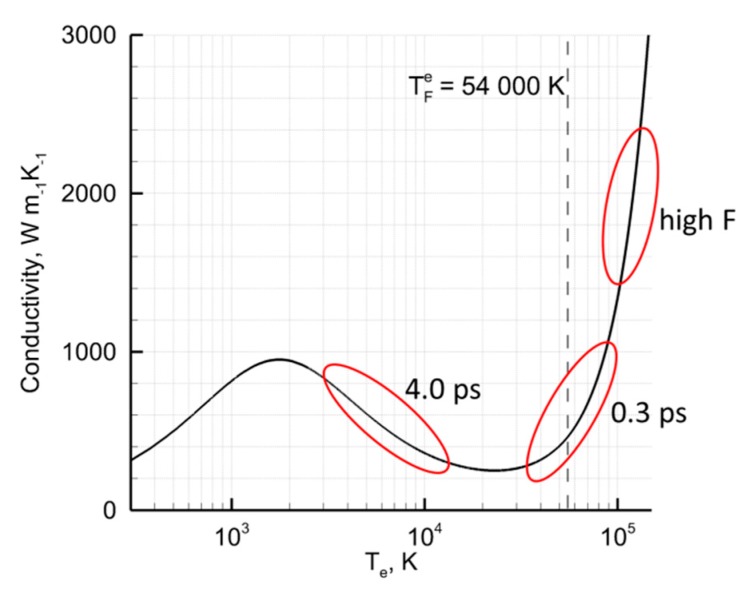
The electron conductivity function is calculated and plotted versus electronic temperature values [48]. The Fermi temperature is indicated by the vertical dashed line. The characteristic values of the electronic temperatures for the corresponding simulations, shown in Figure 3, are indicated in the red ovals for 0.3 ps and 4.0 pulse durations. The oval “high F” indicates the characteristic values for the conductivity function at high fluencies.

The obtained difference, however, can be also explained by dissipation of the laser-deposited energy through different channels: the electron–phonon coupling and the electron heat conductivity. It was shown in [46,47] that depending on the irradiation conditions one or another channel can become dominating, or both channels can be active. For the shorter pulse, the electronic temperature dynamically changes in a wide range, covering three characteristic dependences of electronic conductivity function k_e_: k ~ T_e_ for T_e_ up to 10,000 K, k_e_ ~ T_e_/(AT_ph_ + BT_e_^2^) for T_e_ up to 40,000 K, when an electron–electron collision causes its decay, and like plasma conductivity k_e_ ~ T_e_^5/2^ when T_e_ is in the range of Fermi temperature (54,000 K for Au) and above [48], as shown in Figure 4.

Initially, as the Gaussian laser pulse of 0.3 ps is being absorbed by the electronic system, its temperature scales linearly, which results in effective laser energy penetration to the deep bulk of material and strong temperature and pressure gradients are established over the distance of ~250 nm from the surface. Because of the fast electron–phonon equilibration time, the heating rate therefore exceeds the mechanical relaxation rate over this distance and high compressive stresses are built up inside the target. The laser heating process under such conditions are frequently referred to as the heating under internal stress confinement [49], and, provided that the absorbed energy was high enough, results in the onset of the spallation mechanism of the ablation process. The spallation is accompanied by the relaxation pressure wave causing the nucleation of voids inside the bulk of material in the proximity of the surface that grow and coalesce eventually disrupting the material mechanically. Therefore, the target damage as a result of spallation has more pronounced photomechanical character resulting in the ejection of large chunks of the molten metal or large droplets.

However, the high intensity developed during the Gaussian pulse of 0.3 ps results in the temperature of electrons rising to the range where conductivity function has a decay behavior and only later scaling as k_e_ ~ T_e_^5/2^, see Figure 4. The decay of thee conductivity function shuts off the corresponding channel of the laser-deposited energy dissipation and the absorbed energy is efficiently transferred to the phonon vibrations (due to the electron–phonon coupling mechanism), without penetration to the deeper bulk of the target. Such conditions are referred to the case of thermal confinement and, providing that the absorbed fluence was high enough, results in the temperature of the target surface reaching the critical values (~7000 K for Au). The subsequent ablation process is driven by the explosive boiling mechanism [49], and results in the generation of small clusters and vapor.

As it can be seen from Figure 3a, both mechanisms (the mechanical and thermal damages) are accompanying the ablation of the irradiated target at the pulse duration of 0.3 ps. Up to the distance of 250 ns below the surface, it is under the spallation process during with the formation of voids and their growth takes on a massive character. The high generated electronic temperatures on the surface, however, trigger the thermal damage mechanism and the ejection of the material is provided via efficient vaporization and nucleation of small (~5 nm) NPs. Subsequently, the evolution spallation surface can undergo to Rayleigh–Taylor instability and additional formation of large (~50 nm) particles

The longer pulse, on the other hand, does not generate high values of the electronic temperature, but rather values, where the electronic conductivity function scales linearly in the beginning of the pulse and decays, when it reaches its maximum. The developed conditions, therefore, results in efficient penetration of the laser-deposited energy in the deeper part of the material and establishing of the internal stress confinement regime, Figure 3c. The generated temperatures of the electrons, however, do not reach high values and the deposited laser energy dissipation channel via the electron–phonon coupling can not induce the lattice temperatures significantly above the melting point. The induced target damage for the case of 4.0 ps laser pulse, therefore, has a pure mechanical character, without the ejection of the small clusters and vapor for nucleation of small (~5 nm) NPs.

The real picture of the ablation processes considered above, however, is usually much more complicated and normally can not be separated into the mechanical confinement or thermal confinement regimes only. As it is in the experiment and in the simulations above, both channels of the laser-deposited energy dissipation and the mechanical or thermal confinement regimes sequentially and dynamically changing as interrelated and competing processes and usually they both are involved into the same experiment at different levels and at different depths below the initial materials surfaces. Obviously, the explosive boiling mechanism of the material removal is more preferable for the NPs fraction generation of the smaller possible size. The mechanical confinement regime, on the other hand is responsible for the material ejection in large chunks or large droplets of the material (spallation). The pulse of 0.3 ps results in a large scale of the electronic temperature change. Thus, while rising to the level where conductivity function scales as k_e_ ~ T_e_^5/2^, it inevitably passes through the region of conductivity function scaling linearly, leading to the establishment of the internal stress confinement, and the region when conductivity function decays that leads to the presence of the thermal confinement regime as well. This is reflected in Figure 3a,b, where the generation of a number of NPs of different size takes place. Therefore, due to at least two different mechanisms involved into the material ejection process, the presence of two different NPs size fractions were expected, and it is also confirmed in the experimental measurements, indicated in Figure 1, where the NPs and their size distribution demonstrate the bimodal behavior. A similar size distribution was detected also in number of the experiments [14,15,31,32,33,34,35] and was also given an elegant explanation in the theoretical work by Zhigilei et al. [37]. Namely, while small NPs are rapidly nucleating in a low density mixture of metal-water region, the formation of the second larger fraction is governed by the Rayleigh–Taylor instability resulting in the growth of the extensive jets at the time scale of hundreds of picoseconds and their consecutive destabilization and decomposition of larger droplets in a cold water environment.

**Figure 5:**
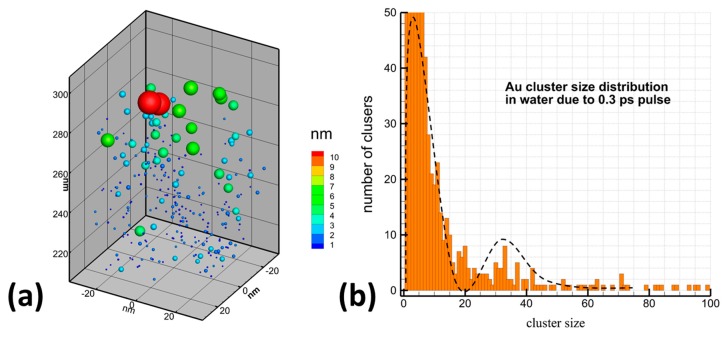
Nanoparticles, generated in water media as a result of 0.3 ps laser pulse interaction with thick Au target. The particles colored and scaled by their corresponding size in nm. The water media and the remaining bulk of Au material are blanked here for the visual analysis (**a**). The nanoparticles’ size distribution, exposing two fractions as it was detected in the experiment. The dashed line is an eye guide only (**b**).

Analyzing Figure 5a, however, where we show the formed NPs as a result of the 0.33 ps laser pulse ablating the Au target in water, one can observe that the smaller and larger fractions of the NPs form at the same time and spatial scale, so that the two distinguished size distribution fractions are clearly seen in Figure 5b. While this fact itself does not exclude two mechanisms of the NPs formation, as suggested by Zhigilei et al. [37], it may also indicate the validity of the explanation based on the interplay of mechanical or thermal damages (or mixture of them) induced with a short laser pulse. In fact, both explanations can complement each other. Indeed, the generation of the significant amount of metal vapor is the result of thermal confinement regime, whereas the establishment of the upward motion of the presurface layer (which can later undergo to Relay–Taylor instability) is a result of the internal stress confinement regime. In other words, the phenomenon of the NPs generation in liquid media can be more or less attributed to all the above-mentioned interrelated, governing mechanisms.

Additionally, the experimental measurements show that one can achieve the generation of a single distribution only. This is possible either by means of changing the irradiation parameters [14,30] or by means of adding different solvents [12,20,28,29]. While the effect of the irradiation parameters were accounted for in both theories of two distributions generation suggested above due to the same MD-TTM approach used [39], both Molecular Dynamics models used for water [43,50] did not include any chemistry since the possibility for the electron density changes, which can lead to the establishment of new type of bonding (chemical reactions, dissociation), was not considered explicitly due to its extremely high computational cost. The change of bimodal NP size distribution due to adding a solvent into the water media can be explained in terms of diffusion process, which is governed not only by the thermophysical conditions (such as temperature, pressure, and density), but also the ability of an atom to diffuse in that media. It was shown in the experiment [20,51] that adding a negligible amount of chemically active molecules can completely suppress the larger size distribution of the NPs. The negligible amount of additive cannot change the heat capacity of the liquid media (which affects the cooling and nucleation process rate of the generated NPs), but can at least lead to the change of diffusivity of the gold atoms in that media due to dissociation processes, not accounted for in the MD simulations.

As it was shown experimentally [14,30,36], one can significantly suppress the smaller NPs distribution, Figure 1, or getting its input negligible as compared to the large distribution when going to the higher values of the incident fluencies. While, the theory suggested in [37] do not address such laser parameters manipulations, the explanation in terms of thermal vs. mechanical damage, given above, indicates clearly that for high fluencies, marked as *high F* in Figure 4, the generated electronic temperatures T_e_ can result in a drastic scaling of conductivity function as k_e_ ~ T_e_^5/2^. The latter will completely take over the dissipation channel through the electron–phonon coupling and the laser-deposited heat will efficiently penetrate to the deeper part of the material with the establishment of strong temperature and pressure gradients over hundreds of nanometer below the surface. For an ultrashort laser pulse irradiation, this corresponds to the case of the internal stress confinement and generation of very large droplets (NPs) as a result of the molten material ejection, which is resulted in the larger NPs mean size, reflected in Figure 1. The modeling of ultrashort laser pulse interactions with gold targets in a water environment under such extreme conditions, however, requires additional computational efforts, and this leaves the intriguing question about the nature of the bimodal size distribution of generated NPs and its manipulations for future investigations.

## 3. Materials and Methods

### 3.1. Fabrication and Characterization of Au Nanoparticles

Au NPs were prepared using methods of femtosecond laser ablation in deionized water [14,15]. Briefly, a gold target (99.99%, GoodFellow Inc., Cambridge, UK) was placed at the bottom of the glass vessel filled with deionized water (18.2 MΩ cm). A 2.3 mm diameter beam from A beam from Yb: KGW femtosecond laser (Amplitude Systems, wavelength 1025 nm, repetition rate 1 kHz, pulse duration 300 fs), was focused with a 75 mm lens on the surface of a target. The target was moved constantly in the focusing plane with a speed of 0.5 mm/s, while keeping the same thickness of the liquid (1 cm) above the target. A high-resolution transmission electron microscopy (HR-TEM) system (JEOL JEM 3010, JEOL Ltd., Croissy-sur-Seine, France) was used to examine size characteristics of laser-synthesized Au NPs.

### 3.2. The Atomistic-Continuum Model MD-TTM

The model consists of two parts: the atomic motion is described within the Molecular Dynamics (MD) approach at atomic precision, whereas in continuum the effect of conduction band electrons is accounted for via their temperature dynamics with the help of the Two Temperature Model (TTM) [52]. The essential concepts of such combined MD-TTM model and its applications to study the evolution of metallic solids excited by an ultrashort laser pulse are described in details in Reference [39]. Thus, the combined model can address the kinetics of fast laser-induced nonequilibrium phase transitions, the laser pulse absorption, fast electron heat conduction, and strong electron–phonon nonequilibrium, induced with a short laser pulse. The combined MD-TTM model was successfully used in studying short pulse laser induced melting, spallation, and ablation of thin and thick metal targets [49,53,54]. Based on the developed approach, the MD-TTM model was further modified for multiprocessing mode with the Message Passing Interface (MPI) algorithm for modeling of laser-induced nanostructuring of metals on the experimental scale [40,55,56]. The performance of the combined MD-TTM method allowed simulating the above system consisting of 185,000,000 atoms over 400 processor cores at Lichtenberg Super Computer Facility TU-Darmstadt and advance for 50 ps of the experimental time per day.

## 4. Conclusions

We developed a framework based on the combination of Molecular Dynamics (MD) and Two Temperature Model (TTM) approaches, to characterize the growth of metal nanoparticles under ultrashort laser ablation from a solid target in water ambient. The model was applied for simulation of the process of bimodal NPs size distribution and analyzing the obtained results we proposed an alternative explanation of the mechanisms responsible for the NPs generation and segregation in water environment. The proposed mechanism allows one to manipulate with the NPs size distribution via the irradiation parameters, which was confirmed experimentally. It was indicated that depending the incident fluence or the pulse duration, one can trigger thermal or mechanical character of the irradiated target damage (or mixture of them). Correspondingly, the activated one or another regime of the ablation process will result in a massive generation of one or another NPs size. This theory, however, do not exclude other explanations on the nature of bimodal NPs size distribution, and still cannot address the effect of chemical additives that can also alter the NPs distribution.
